# Murine sca1/flk1-positive cells are not endothelial progenitor cells, but B2 lymphocytes

**DOI:** 10.1007/s00395-020-0774-6

**Published:** 2020-01-24

**Authors:** Eva Steffen, Wolfgang Bernd Edziu Mayer von Wittgenstein, Marie Hennig, Sven Thomas Niepmann, Andreas Zietzer, Nikos Werner, Tienush Rassaf, Georg Nickenig, Sven Wassmann, Sebastian Zimmer, Martin Steinmetz

**Affiliations:** 1 Herzzentrum Bonn, Medizinische Klinik und Poliklinik II, Universitätsklinikum Bonn, Venusberg Campus 1, 53127 Bonn, Germany; 2 Westdeutsches Herz- und Gefäßzentrum, Klinik für Kardiologie und Angiologie, Universitätsklinikum Essen, Essen, Germany; 3 Cardiology Pasing, Munich, Germany; 4 University of the Saarland, Homburg, Saar Germany; 5 Krankenhaus der Barmherzigen Brüder, Innere Medizin III, Trier, Germany

**Keywords:** Endothelial progenitor cells, B Lymphocytes, Atherosclerosis, Endothelial dysfunction, Endothelial regeneration

## Abstract

Circulating sca1^+^/flk1^+^ cells are hypothesized to be endothelial progenitor cells (EPCs) in mice that contribute to atheroprotection by replacing dysfunctional endothelial cells. Decreased numbers of circulating sca1^+^/flk1^+^ cells correlate with increased atherosclerotic lesions and impaired reendothelialization upon electric injury of the common carotid artery. However, legitimate doubts remain about the identity of the putative EPCs and their contribution to endothelial restoration. Hence, our study aimed to establish a phenotype for sca1^+^/flk1^+^ cells to gain a better understanding of their role in atherosclerotic disease. In wild-type mice, sca1^+^/flk1^+^ cells were mobilized into the peripheral circulation by granulocyte-colony stimulating factor (G-CSF) treatment and this movement correlated with improved endothelial regeneration upon carotid artery injury. Multicolor flow cytometry analysis revealed that sca1^+^/flk1^+^ cells predominantly co-expressed surface markers of conventional B cells (B2 cells). In RAG2-deficient mice and upon B2 cell depletion, sca1^+^/flk1^+^ cells were fully depleted. In the absence of monocytes, sca1^+^/flk1^+^ cell levels were unchanged. A PCR array focused on cell surface markers and next-generation sequencing (NGS) of purified sca1^+^/flk1^+^ cells confirmed their phenotype to be predominantly that of B cells. Finally, the depletion of B2 cells, including sca1^+^/flk1^+^ cells, in G-CSF-treated wild-type mice partly abolished the endothelial regenerating effect of G-CSF, indicating an atheroprotective role for sca1^+^/flk1^+^ B2 cells. In summary, we characterized sca1^+^/flk1^+^ cells as a subset of predominantly B2 cells, which are apparently involved in endothelial regeneration.

## Introduction

Damage of the endothelium is a main feature of a variety of vascular diseases including atherosclerosis or upon percutaneous transluminal angioplasty. Such damage leads to endothelial dysfunction and finally the deterioration of the endothelial cells, which can further result in plaque progression or restenosis following interventional treatment [5, 12]. The renewal of endothelial cells is crucial to restrain or even prevent the process of atherosclerosis and restenosis. It has been hypothesized that the endothelium is restored in loco by proliferation of neighboring endothelial cells (ECs). This paradigm has been challenged by Asahara and a plethora of later studies, which proposed that circulating endothelial progenitor cells (EPCs) significantly contribute to reendothelialization [1]. Those putative EPCs have been associated with endothelial restoration and have been shown to be inversely correlated with atherogenesis in humans and mice [8, 25, 28, 33, 35, 36, 38].

However, the identification of EPCs has been challenging due to the fact that EPCs do not express a specific surface marker and do not demonstrate characteristics that allow for their definitive identification. A variety of surface marker combinations and cell types have been used for the investigation of EPCs, although these markers are also expressed on other cell subsets such as hematopoietic cells or mature endothelial cells [6, 13, 15, 29]. Thus, studies investigating EPCs are hard to compare and present conflicting results [42].

In one study, injected sca1^+^/flk1^+^ cells were detected in the endothelial layer of the neointima following femoral artery injury [40]. In ApoE^−/−^ mice that have received transplanted bone marrow from eGFP transgenic mice, only 1 cell out of almost 3000 cells analyzed in carotid bifurcation plaques originated from the bone marrow [9]. In a murine model of hindlimb ischemia, bone-marrow-derived stem cells were not incorporated into the vascular wall. Instead, they featured a supportive, paracrine function that supported angiogenesis [44]. We found in previous studies that sca1^+^/flk1^+^ cells were mobilized from the bone marrow and correlated with vascular health [27, 28, 30].

As a consequence of these puzzling results, the identity and function of EPCs as well as their role in endothelial health are still under heavy debate [13, 15, 21].

The identification of the cellular origins of putative EPCs and their pathophysiological involvement in vascular damage and regeneration are pivotal to develop efficient approaches for their use, with a reliable, therapeutic benefit.

In this study, we sought to scrutinize the identity of sca1^+^/flk1^+^ cells, to amend the current hypothesis of vascular regeneration by circulating cells.

## Materials and methods

### Animals and treatment regimen

All experiments were performed in accordance with institutional guidelines and the German animal protection law. 12-week-old male and female C57/Bl6J mice (Charles River, Wilmington, USA) and 12-week-old female RAG2-deficient mice (The Jackson Laboratory, Bar Harbor, USA) were used in this study. All animals were maintained in a 12-h dark/light cycle and received water and food ad libitum. Medical treatment, B2 cell depletion and monocyte depletion were performed as indicated below.

For mobilization experiments, 12-week-old C57/Bl6J mice were randomly assigned to two groups and received G-CSF (0.05 µg/g body weight; Neupogen^®^, AMGEN, Thousand Oaks, USA) or PBS once per day for 10 days. All substances were administrated intraperitoneally.

For B2 cell depletion, 12-week-old male C57/Bl6J mice received an intraperitoneal injection of 50 µg of an anti-CD20 antibody (Ultra-LEAF™ Purified anti-mouse CD20 Antibody, clone SA271G2, Biolegend, San Diego, USA) or an isotype identical control (Ultra-LEAF™ Purified Rat IgG2b, k Isotype Ctrl Antibody, clone RTK4530, Biolegend, San Diego, USA). After 3 days, B2 lymphocytes were almost completely depleted. The application of the antibody was repeated after 7 days. For monocyte depletion, 12-week-old male C57/Bl6J mice received an intraperitoneal injection of 200 µl of clodronate-filled liposomes (clodronate liposomes, 5 mg/ml, Liposoma B.V., Amsterdam, the Netherlands) or PBS-filled control liposomes (Liposoma B.V., Amsterdam, the Netherlands). 24 h after injection, a nearly complete depletion of classical and non-classical monocytes was achieved. The liposome injection was repeated every other day. B2 cell and monocyte depletions were confirmed by flow cytometry on the day of the carotid artery denudation and on the day of killing. Animals without sufficient B2 cell or monocyte depletion were excluded from the study.

### Carotid artery denudation and assessment of reendothelialization

For assessment of reendothelialization (i.e., endothelial regeneration), mice were anesthetized and the left common carotid artery was subjected to an electric injury starting at the bifurcation point and continuing 4 mm in the proximal direction as previously described [28, 45]. Five days after injury, mice were killed and 50 µl of Evans blue solution (Sigma, Kawasaki, Japan) was applied through the left ventricle. One minute after injection, the vascular system was flushed with 4 ml of NaCl 0.9% to remove excess Evans blue dye from the vasculature. Subsequently, the harvested carotid arteries were mounted on a microscope slide. Blue areas were considered to be denudated and/or damaged, and unstained areas as healthy and/or regenerated. Measurements were computer based and executed by a blinded investigator (AxioVision version 4.5.0 software, Zeiss, Jena, Germany).

### Flow cytometry

Flow cytometry analysis was performed as previously described [28, 30]. Samples were measured on a FACSCalibur or FACSCanto II (BD Bioscience, Franklin Lakes, USA) and analyzed with FlowJo (Tree Star, Ashland, USA). Dead cells were identified by using a Zombie Aqua™ Fixable Viability Kit (Biolegend, San Diego USA). Doublets were excluded in every analysis. Samples were stained with anti-sca1-APC (R&D Systems, Minneapolis, USA) anti-flk-1-PE (BD Bioscience, Franklin Lakes, USA), anti-B220-APC (BD Bioscience, Franklin Lakes, USA), anti-IgM-Per-CP/Cy5.5 (BD Bioscience, Franklin Lakes, USA), anti-CD11b-APC/Cy7 (BD Bioscience, Franklin Lakes, USA), anti-CD5-PE (BD Bioscience, Franklin Lakes, USA), anti-sca1-FITC (BD Bioscience, Franklin Lakes, USA), anti-flk1-PE-Cy7 (BD Bioscience, Franklin Lakes, USA), anti-CD3-FITC (BD Bioscience, Franklin Lakes, USA), anti-CD4-PE/Cy7 (eBioscience, Waltham, USA), anti-CD8-APC (Biolegend, San Diego, USA), anti-sca1-PerCP/Cy5.5 (eBioscience, Waltham, USA), anti-CD11b-PE (BD Bioscience, Franklin Lakes, USA), anti-CD115-APC (Biolegend, San Diego USA), anti-Gr1-FITC (BD Bioscience, Franklin Lakes, USA) and anti-CD45 FITC (BD Bioscience, Franklin Lakes, USA).

### Fluorescence-activated cell sorting

Spleen cells were stained with anti-CD45-FITC (BD Bioscience, Franklin Lakes, USA), anti-sca1-BV421 (BD Bioscience, Franklin Lakes, USA), anti-flk1-PE-Cy7 (BD Bioscience, Franklin Lakes, USA), anti-B220-APC (BD Bioscience, Franklin Lakes, USA), anti-IgM-Per-CP/Cy5.5 (BD Bioscience, Franklin Lakes, USA), anti-CD11b-APC/Cy7 (BD Bioscience, Franklin Lakes, USA) and anti-CD5-PE (BD Bioscience, Franklin Lakes, USA). Dead cells were identified by using a Zombie Aqua™ Fixable Viability Kit (Biolegend, San Diego USA) and doublets were excluded.

The following fractions were sorted by using flow cytometry (FACSAria, BD): CD45^+^, sca1^+^/flk1^+^ (sca1^+^/flk1^+^ cells); CD45^+^, B220^+^/IgM^+^, CD11b^−^/CD5^−^ (B2 cells); CD45^+^/sca1^−^/flk1^−^, B220^+^/IgM^+^, CD11b^−^/CD5^−^ (sca1^−^/flk1^−^ B2 cells).

### Mouse surface marker PCR array and RNA-sequencing

To confirm the identity of sca1^+^/flk1^+^ putative EPCs, a mouse surface marker PCR array of isolated sca1^+^/flk1^+^ putative EPCs, conventional B2 cells, and sca1/flk1-depleted B2 cells was performed (Cell Surface Markers PCR Array, Qiagen, Hilden, Germany). The PCR array was used in accordance with the manufacturer’s instructions. Unsorted spleen cells were used as controls. To get further insights into the identity of sca1^+^/flk1^+^ putative EPCs, RNA sequencing was performed on the sorted sca1^+^/flk1^+^ putative EPCs. Fluorescence-activated cell sorting was performed as indicated above. Sorted B2 cells and sca1^−^/flk1^−^ B2 cells were used as controls.

### Statistical analysis

The results are presented as means ± SEM and were analyzed with the Student’s *t* test. Values of *p* ≤ 0.05 were considered significant.

## Results

### Increased level of sca1^+^/flk1^+^ cells correlates with enhanced endothelial regeneration

**Fig. 1:**
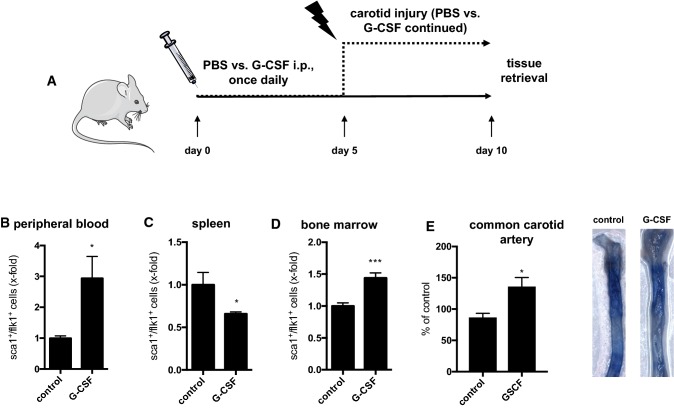
sca1^+^/flk1^+^ cells are pharmacologically increased by G-CSF in the bone marrow and peripheral circulation and correlate with improved endothelial regeneration in the common carotid artery upon injury. **a** Experimental design to increase sca1^+^/flk1^+^ cells by injecting recombinant G-CSF. **b**–**d** Quantification of sca1^+^/flk1^+^ cells in peripheral blood, spleen, and bone marrow after 10 days of G-CSF treatment (*n* = 8–10, *p ≤ 0.05, *** ≤ 0.001). **e** Endothelial regeneration following electric injury of the common carotid artery during treatment with PBS (control) or G-CSF (*n* = 4–5; *p ≤ 0.05)

One putative, mouse-specific subset of circulating EPCs is defined as sca1^+^/flk1^+^ cells. This subset has been repeatedly shown to be associated with vascular health and can be increased with various drugs. Furthermore, the increase of sca1^+^/flk1^+^ cells correlates with improved endothelial dysfunction [29]. In a first series of experiments, we administered recombinant granulocyte-colony stimulating factor (G-CSF, Fig. 1a), which is known to mobilize cells of the endogenous stem cell and progenitor cell pools. In two independent experiments, we found elevated levels of sca1^+^/flk1^+^ cells in peripheral blood and bone marrow, but a reduction of sca1^+^/flk1^+^ cells among splenocytes (Fig. 1b–d). We also inflicted an electric injury of the common carotid artery on some of the mice to assess endothelial regeneration. G-CSF treatment led to increased sca1^+^/flk1^+^ cells in the peripheral blood and was associated with improved restoration of the endothelium in the area of damage (Fig. 1e).

### Hematopoietic surface markers of lymphocytes and monocytes/macrophages are co-expressed on sca1^+^/flk1^+^ cells

**Fig. 2:**
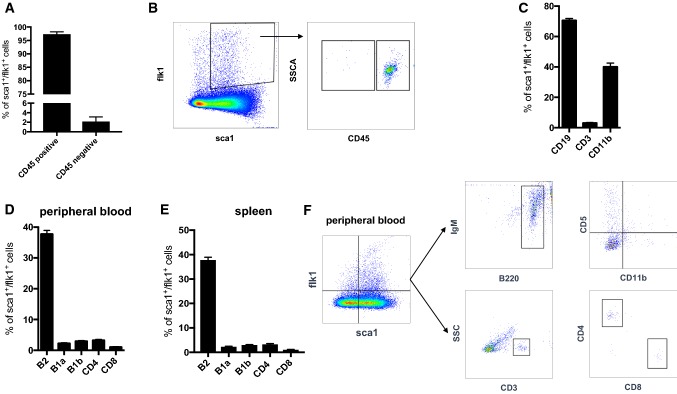
sca1^+^/flk1^+^ cells are hematopoietic cells with lymphocyte and monocyte surface markers. **a** Quantification of CD45-positive and -negative sca1^+^/flk1^+^ cells in peripheral blood in 12 weeks old male C57/Bl6J mice (*n* = 5). **b** Representative images of CD45 expression of sca1^+^/flk1^+^ cells in peripheral blood of 12-week-old male C57/Bl6J mice. **c** sca1^+^/flk1^+^ cells mainly express the lymphocyte markers CD19 and CD3, and CD11b. **d** Quantification of B- and T-cell subsets among sca1^+^/flk1^+^ cells in peripheral blood, and in (**e** spleen (*n* = 5–7). **f** Gating strategy for the investigation of B- and T-cell markers on sca1^+^/flk1^+^ cells

**Fig. 3:**
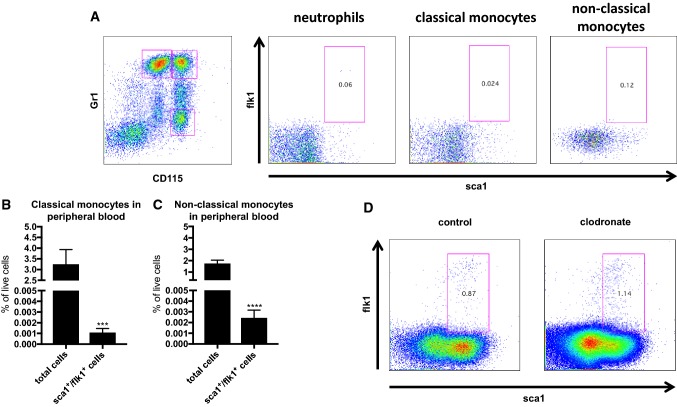
sca1^+^/flk1^+^ cells are not monocytes or neutrophils. **a** Gating strategy to investigate sca1^+^/flk1^+^ cells among neutrophils (CD11b^+^/Gr1^high^/CD115^neg^), classical (CD11b^+^/Gr1^high^/CD115^pos^), and non-classical (CD11b^+^/Gr1^low^/CD115^pos^) monocytes. Expression of **b** classical and **c** non-classical monocyte markers is significantly decreased in sca1^+^/flk1^+^ cells (*n* = 9–10; ***p ≤ 0.001, ****p ≤ 0.0001). **d** Representative flow cytometry images of sca1^+^/flk1^+^ cells after monocyte depletion in C57/Bl6J mice

**Fig. 4:**
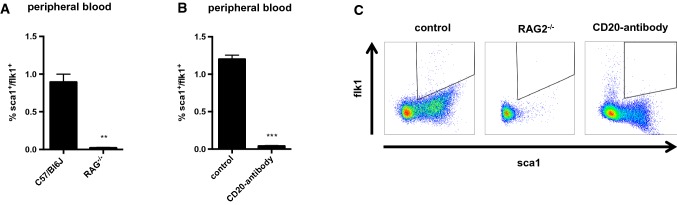
sca1^+^/flk1^+^ cells are almost undetectable in B- and T-cell-deficient RAG2^−/−^ mice or following B2 cell depletion. **a** sca1^+^/flk1^+^ cells are scarcely detectable in the peripheral blood of RAG2-deficient (RAG2^−/−^) mice. **b** The depletion of B2 cells with anti-CD20 antibody leads to a dramatic drop of sca1^+^/flk1^+^ cells. **c** Flow cytometry images of sca1^+^/flk1^+^ cells in wild-type mice, RAG2^−/−^ mice, and anti-CD20-treated mice lacking B2 cells (*n* = 7–11, **p ≤ 0.01, ***p ≤ 0.001)

Flow cytometry analysis of peripheral blood samples from wild-type mice showed that sca1^+^/flk1^+^ cells are also positive for CD45 and approximately 70% of the cells co-express the B cell surface antigen B4 (CD19) **(**Fig. 2a, b**)**. There is also a low level of the T-cell co-receptor (CD3) expressed by the cells and approximately 40% express the macrophage-1 antigen (Mac1 or CD11b, Fig. 2c). When we scrutinized different lymphocyte subsets, sca1^+^/flk1^+^ cells appeared to be mostly the conventional B2-type (B220^+^/IgM^+^, CD11b^−^/CD5^−^) B cells, but there were also some B1a (B220^+^/IgM^+^, CD11b^+^/CD5^+^) and B1b (B220^+^/IgM^+^, CD11b^+^/CD5^−^) cells as well as T-helper (CD4) and cytotoxic (CD8) T cells (Fig. 2d). The analysis of spleen-derived sca1^+^/flk1^+^ cells showed the same distribution pattern of B- and T-cell subsets as in peripheral blood **(**Fig. 2e**).** When we investigated sca1^+^/flk1^+^ cells for a possible monocyte/macrophage identity, we found that classical and non-classical monocytes as well as neutrophils did not express significant levels of sca1 and flk1 (Fig. 3a–c). Furthermore, when we depleted the level of monocytes in the blood by using clodronate, the level of sca1^+^/flk1^+^ cells remained unaffected (Fig. 3d). Using a similar approach, we next investigated the level of sca1^+^/flk1^+^ cells in mice with a lymphocyte deficiency. In RAG2^−/−^ mice, which lack of functional T and B cells, sca1^+^/flk1^+^ cells were almost completely undetectable (Fig. 4a, c). When we depleted the blood of B2 cells by using an anti-CD20 antibody, we observed a similar reduction in the number of sca1^+^/flk1^+^ cells (Fig. 4b, c).

### Expression analyses of cell surface markers on purified sca1^+^/flk1^+^ cells confirm a heterogeneous leukocyte identity with a predominant B cell phenotype

**Fig. 5:**
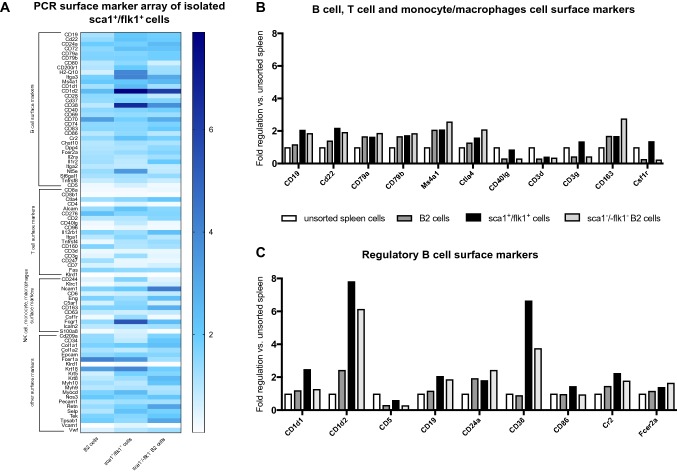
Expression of B cell surface markers is similar in purified sca1^+^/flk1^+^ cells compared to conventional B2 cells and sca1^−^/flk1^−^ B2 cells. **a** Heat map of surface-marker mRNA expression in spleen-derived sca1^+^/flk1^+^ cells, B2 cells and sca1^−^/flk1^−^ B2 cells. Cells were isolated by fluorescence activated cell sorting and compared to normal spleen cells. **b** Expression of B cell, T cell, and monocyte/macrophage cell-surface markers and **c** regulatory B cell markers in sca1^+^/flk1^+^ cells, B2 cells, sca1^−^/flk1^−^ B2 cells, and unsorted spleen cells. Samples of isolated sca1^+^/flk1^+^, B2 cells, and sca1^−^/flk1^−^ B2 cells were pooled from *n* = 10

**Fig. 6:**
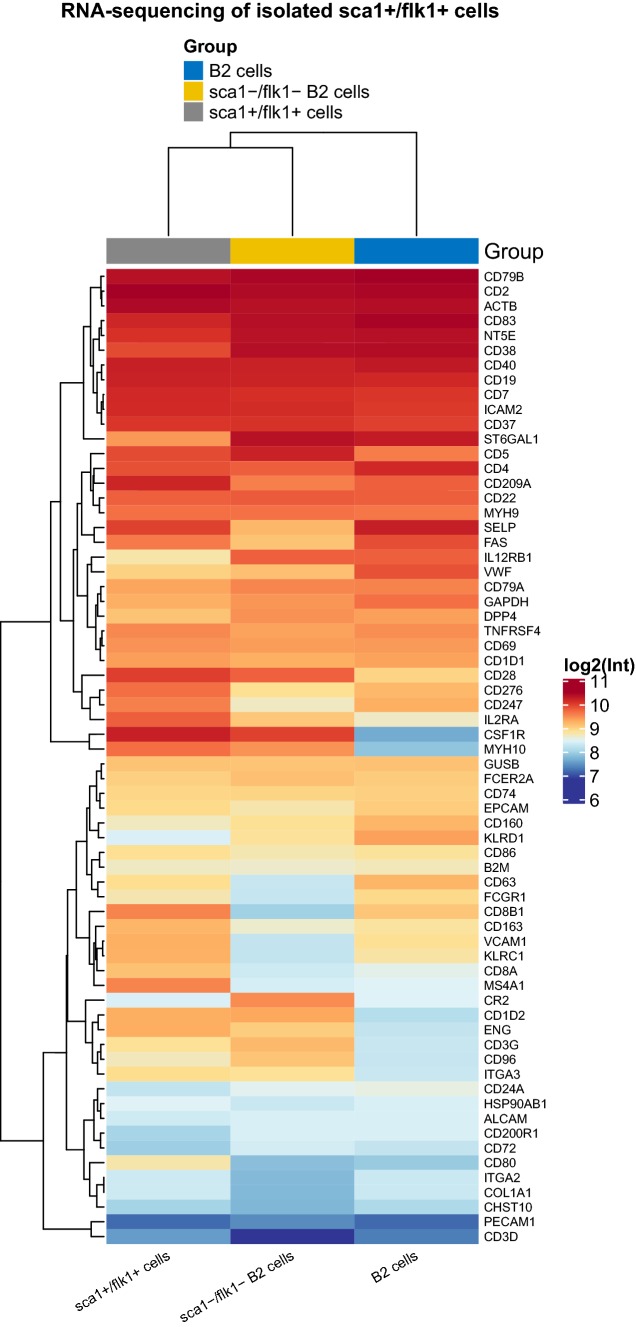
Analysis of sca1^+^/flk1^+^ cells by RNA sequencing confirms their predominant B cell-like phenotype. Heat map of surface marker mRNA expression in spleen-derived sca1^+^/flk1^+^ cells, B2 cells, sca1^−^/flk1^−^ B2 cells, and unsorted spleen cells is shown. Samples were analyzed by using RNA sequencing. Samples of isolated sca1^+^/flk1^+^, B2 cells, and sca1^−^/flk1^−^ B2 cells were pooled from *n* = 10

To further dissect the cellular identity of sca1^+^/flk1^+^ cells, we isolated this subset of cells from murine spleens by fluorescence-activated cell sorting. Using a cell surface marker expression analysis of the cellular mRNA profile, we found similar expression levels of CD19, CD22, CD79a, CD79b, and MS4a1 mRNA in sca1^+^/flk1^+^ cells compared to B2 cells and sca1^−^/flk1^−^ B2 cells, which are exclusive to B lymphocytes. Furthermore, T-cell markers such as Ctla4, CD40lg, and CD3d appeared to be downregulated in these cells **(**Fig. 5a, b**)**, compared to the controls. However, we also detected a few markers of T cells, such as CD3g, and monocyte/macrophages, such as CD163 or Csf1r, to be upregulated in sca1^+^/flk1^+^ cells **(**Fig. 5a, b). When we analyzed the gene expression of sca1^+^/flk1^+^ cells with respect to specific B cell subsets, we found a noticeable upregulation of surface markers that are characteristic for regulatory B cells, such as CD1, CD86, or Cr2 **(**Fig. 5c**)**. Furthermore, CD38 appeared to be highly upregulated in sca1^+^/flk1^+^ cells, which is a surface marker on human regulatory B lymphocytes. To scrutinize the B2 cell-like phenotype of sca1^+^/flk1^+^ cells and to identify possible key regulated genes, sca1^+^/flk1^+^ cells, B2 cells, and sca1^−^/flk1^−^ B2 cells were analyzed by RNA sequencing. In line with our previous results, we found a similar gene expression profile in the three groups, especially with regard to the expression of B cell surface markers **(**Fig. 6**)**.

### The depletion of B cells including sca1^+^/flk1^+^ cells leads to a decline in endothelial regeneration in G-CSF treated wild-type mice

**Fig. 7:**
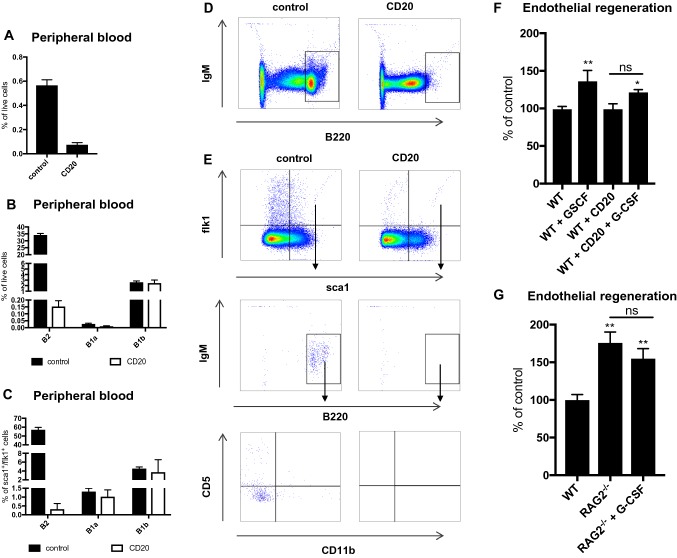
sca1^+^/flk1^+^ cells are mandatory for endothelial regeneration after G-CSF treatment. **a** sca1^+^/flk1^+^ cells in peripheral blood after B2 cell depletion (*n* = 7–10). **b** Subsets of B cells in peripheral blood after administration of G-CSF and B2 cell depletion (*n* = 7–10). **c** Subsets of sca1^+^/flk1^+^ B cells in peripheral blood after administration of G-CSF and B2 cell depletion (*n* = 7–10). **d** Representative image of B2 cell depletion after administration of the anti-CD20 antibody. **e** The gating strategy used to analyze sca1^+^/flk1^+^ B cell subsets in peripheral blood after B2 cell depletion. **f** Endothelial regeneration after administration of G-CSF, administration of G-CSF and B2 cell depletion, or B2 cell depletion alone (*n* = 5–14, *p ≤ 0.05, **p ≤ 0.01, *ns* not significant). **g** Endothelial regeneration in RAG2^−/−^ mice at baseline and after administration of G-CSF (*n* = 5, **p ≤ 0.01)

Finally, we further investigated whether B2- and sca1^+^/flk1^+^ cells may directly impact endothelial regeneration, which had been so far hypothesized by their correlation with endothelial health. We tested reendothelialization after antibody-mediated depletion of B2 cells including sca1^+^/flk1^+^ cells, as well as in RAG2-deficient mice, with and without concomitantly administered G-CSF **(**Fig. 7**)**. The depletion of B2 cells and sca1^+^/flk1^+^ cells was verified by flow cytometry in each experiment **(**Fig. 7a–e**)**. Depletion of sca1^+^/flk1^+^ cells alone did not affect endothelial regeneration under basal, unstimulated conditions **(**Fig. 7f**)**. However, when mice were given G-CSF, we found a decline in the endothelial regeneration in the mice that lacked B2- and sca1^+^/flk1^+^ cells compared to their littermate controls **(**Fig. 7f). In RAG2^−/−^ mice, endothelial regeneration appeared to be enhanced at baseline compared to wild-type mice, which can be explained by the absence of pro- and antiatherogenic influences due to their immunodeficiency [20]. However, the administration of G-CSF did not further improve endothelial regeneration, indicating that the subset of sca1^+^/flk1^+^ cells, or at least B2 cells, is mandatory for the endothelial-regenerating effect of G-CSF (Fig. 7g**)**.

## Discussion

Since they were first described in 1997 by Asahara et al., a multitude of studies have investigated the impact of putative EPCs on vascular regeneration and atherosclerosis [1, 15, 29, 35, 37, 42]. Due to legitimate doubts concerning their identity and function, our study aimed to scrutinize sca1^+^/flk1^+^ cells, which had thus far been considered to be EPCs [4, 7, 9, 13, 21, 32].

To demonstrate the higher potential for endothelial regeneration in mice with higher circulating levels of sca1^+^/flk1^+^ cells, the animals were treated with G-CSF, a well-established mobilizing agent of putative EPCs [17, 19]. As expected, G-CSF treatment led to elevated levels of circulating sca1^+^/flk1^+^ cells in the peripheral blood and an enhancement of endothelial regeneration following electric injury of the common carotid artery, which is in line with previous studies by ours and other groups. One study demonstrated that the application of G-CSF leads to accelerated endothelial regeneration and neointimal formation after wire-mediated vascular injury of the femoral artery in C57/Bl6J mice [43]. Studies from our group have shown that mobilization of sca1^+^/flk1^+^ cells with different mobilizing agents is associated with an increase in endothelial regeneration, whereas reduced levels of these cells correlate with an impairment of endothelial regeneration upon electric injury of the common carotid artery. Moreover, we demonstrated in a hindlimb in situ perfusion model that sca1^+^/flk1^+^ cells are at least partially mobilized from the bone marrow and that the ability to mobilize these cells declines with age and the severity of atherosclerosis [27, 28, 30]. When we analyzed sca1^+^/flk1^+^ cells with respect to their identity, we found that the majority of sca1^+^/flk1^+^ cells expressed CD45. This result has been reported before by Wheat et al. who studied the effects of acrolein inhalation on sca1^+^/flk1^+^ cells in mice and reported that these cells were positive for CD45 [39]. We analyzed hematopoietic lineage markers, which revealed the co-expression of lymphocyte and monocyte/macrophage markers on sca1^+^/flk1^+^ cells, with a preponderance of conventional B2 lymphocytes. To confirm the predominant B cell-like phenotype of sca1^+^/flk1^+^ cells, we used flow cytometry-based cell sorting and analyzed their intracellular transcripts by mRNA profiling and RNA sequencing. We detected a similar expression of B cell surface markers in sca1^+^/flk1^+^ cells compared to conventional B2 cells and sca1/flk1-depleted B2 cells. We also detected an upregulation of scattered T-cell and monocyte/macrophage markers, which strengthens our flow cytometry data. However, there was a striking dominance of B2 cell markers. Finally, the depletion of lymphocytes in RAG2^−/−^ mice, and especially B2 cell depletion with anti-CD20, was associated with a concomitant, total depletion of sca1^+^/flk1^+^ cells, whereas monocyte depletion did not affect sca1^+^/flk1^+^ cells in a significant way.

B cells are important modulators of atherosclerotic disease that act by antibody secretion, production of cytokines or T-cell regulation (see reviews [23, 24]). The subset of B2 cells is a heterogeneous population, comprising follicular, marginal zone, and regulatory B cells with different impacts on lesion development [18]. Whereas Kyaw et al. suggested an overall proatherogenic role for B2 cells, Nus et al. showed that marginal zone B cells protect from lesion development by inhibiting a proatherogenic response of T-follicular helper cells [11, 16]. Strom et al. identified a lymph node-derived subset of regulatory B2 cells that reduced neointima formation by secreting IL-10 [31]. The relevance of IL-10 production was also highlighted by Ponnuswamy et al., who demonstrated a synergy between angiotensin II and BAFF, resulting in an induction of atheroprotective regulatory B2 cells [18]. Interestingly, we found several surface markers that are also expressed on regulatory B cells, such as CD1, Cr2, or CD86, to be upregulated in sca1^+^/flk1^+^ cells [14, 22]. Furthermore, we found a strong upregulation of CD38. CD38 is a transmembrane receptor and ectoenzyme, which is found on several B cell subsets and is involved in apoptosis, cell differentiation, proliferation, and activation [34]. In humans, regulatory CD19^+^,CD24^hi^,CD38^hi^ B cells have been shown to inhibit the production of proinflammatory cytokines by CD4^+^ T cells [2]. A murine subset of regulatory B cells that express CD38 has not yet been described [14, 22].

Having identified sca1^+^/flk1^+^ cells as mainly B2 cells, we wanted to investigate their impact on endothelial regeneration. Therefore, we tested whether the depletion of B2 cells and sca1^+^/flk1^+^ cells would affect reendothelialization. In mice treated with an anti-CD20 antibody, the benefit on reendothelialization from G-CSF treatment declined. In RAG2^−/−^ mice, which lack mature B and T lymphocytes as well as sca1^+^/flk1^+^ cells, G-CSF treatment had no effect on endothelial regeneration. These data suggest that G-CSF-induced reendothelialization depends on B2 cells, which include sca1^+^/flk1^+^ cells.

One possible mechanism could be an enhanced mobilization of protective sca1^+^/flk1^+^ B2 cells into the peripheral circulation. Studies from cancer research have already demonstrated the angiogenic potential of B cells [3]. B cells are able to promote angiogenesis in lymph nodes by paracrine secretion of VEGF [26]. STAT3 activation in B cells contributes to tumor progression by enhancing angiogenesis. Co-culturing endothelial cells with naïve spleen-derived B cells was associated with a significant increase in tube formation [41]. In our study, we found surface markers of regulatory B cells upregulated in sca1^+^/flk1^+^ cells. One hypothesis is that IL-10 signaling is a possible mechanism by which sca1^+^/flk1^+^ cells contribute to endothelial regeneration [22]. Interestingly, it has been reported that G-CSF itself is able to promote angiogenesis [10]. However, since endothelial regeneration declined in G-CSF-treated wild-type mice after B2 cell depletion, our data raise doubts about that mode of action for G-CSF.

One limitation is that anti-CD20-treatment depletes the whole subset of B2 cells including sca1^+^/flk1^+^ cells. However, the focus of our study was to clarify the identity and function of sca1^+^/flk1^+^ cells.

In conclusion, our study shows that sca1^+^/flk1^+^ cells are not EPCs, but lymphocytes instead, comprising mainly B2 lymphocytes. Although they are not true vascular progenitor cells, B cells do contribute to G-CSF-mediated endothelial regeneration. Furthermore, sca1^+^/flk1^+^ B cells were confirmed as a biomarker that correlates with reendothelialization. Their contribution to endothelial regeneration still needs to be elucidated and should be addressed in future studies.
